# ‘Maybe It Is Only in Prison That I Could Change Like This’ The Course of Severe Mental Illnesses During Imprisonment – A Qualitative 3-Year Follow-Up Study From Chile

**DOI:** 10.3389/fpsyg.2020.01208

**Published:** 2020-06-08

**Authors:** Caroline Gabrysch, Carolina Sepúlveda, Carolina Bienzobas, Adrian P. Mundt

**Affiliations:** ^1^Department of Psychiatry and Psychotherapy, Charité Campus Mitte, Charité – Universitätsmedizin Berlin, Berlin, Germany; ^2^Department of Psychiatry and Mental Health, Hospital Clínico Universidad de Chile, Santiago, Chile; ^3^Department of Psychology, Universidad Academia de Humanismo Cristiano, Santiago, Chile; ^4^Medical Faculty, Universidad San Sebastián, Puerto Montt, Chile; ^5^Medical Faculty, Universidad Diego Portales, Santiago, Chile

**Keywords:** prison, severe mental illness, major depression, psychosis, substance use disorders, qualitative

## Abstract

**Background:**

Severe mental illness (SMI) among imprisoned individuals is a global health concern. Quantitative research indicates on average mental health symptom improvements during imprisonment, however, it cannot reflect multifaceted factors influencing the course of SMI. This study aimed to explore the subjective course of SMI during imprisonment and to identify influencing factors.

**Methods:**

The study has a 3-year-follow-up design of imprisoned individuals in Chile. We conducted semi-structured interviews with 10 men and 9 women at follow-up who had either major depression or psychosis (severe mental illnesses) at baseline. We included individuals who deteriorated, remained stable or improved their mental health according to quantitative assessments. We explored the subjective course of their mental health condition during the follow-up period. Qualitative data was transcribed and coded using NVivo Software for quantitative content analysis. Qualitative data was also manually coded and was subsequently analyzed using the thematic analysis method with an inductive approach. We developed the final themes using the results of this analysis in combination with the inclusion criteria.

**Results:**

The mental health of 10 individuals subjectively improved, 6 experienced deteriorations, and 3 did not perceive any change. Good infrastructure, structure/occupation, intrapersonal resources (will to change, spirituality) and supportive relationships were identified as factors improving mental health. Factors deteriorating mental health were identified as bad infrastructure (e.g., no running water and bad housing conditions) and crowding, lack of psychological treatment, exposure to violence, interpersonal stress (conflictive relationships and separation from family), perceived injustice through sentencing, intrapersonal stressors and previous medical conditions.

**Discussion:**

SMI in prison can improve in a supportive environment under certain conditions. These conditions include the improvement of infrastructure (housing and healthcare), the opportunity to work or study, protection from violence during imprisonment, and to develop intrapersonal resources and family relationships. To reduce SMI in prisons the improvement of these conditions should become a priority.

## Introduction

Prison populations have grown worldwide in the past decades, especially in South America (Walmsley, 2018), where the increase of prison populations was associated with the removal of psychiatric beds (Mundt et al., 2015a). Rates of mental disorders in prison populations are high (Fazel and Seewald, 2012) and severe mental illness (SMI) is more prevalent than in the general population (Fazel and Baillargeon, 2011). In recent years, this has also been shown for prison populations in low and middle income countries (LMIC) (Mundt et al., 2013; Andreoli et al., 2014; Baranyi et al., 2019). The disregard of people with mental illness in prisons in LMICs is a global health challenge (Jack et al., 2018). Longitudinal studies using quantitative psychometric methods show that symptoms of SMI in imprisoned people on average improve over time during imprisonment (Walker et al., 2014). Especially imprisoned individuals with previous mental illness and problematic alcohol/drug use might improve in the structured prison environment (Dirkzwager and Nieuwbeerta, 2018).

An important point to consider for this study were possible differences in the definition of the *imprisoned individual with SMI* between the legal system and the psychiatric care system. The legal system distinguishes between individuals with mental disorders (diagnosed by DSM-V or ICD-10) and individuals with mental disease or defect as a legal criterion for the insanity defense that has to be evaluated by a forensic psychiatrist (Giorgi-Guarnieri et al., 2002). Andrew and Bonta propose to define the mentally ill offender as someone who is imprisoned and meets the diagnostic criteria for axis I or axis II disorders of the DSM-V (Andrews and Bonta, 2010). We followed Andrew and Bontas’ definition in our study and selected those who met the diagnostic criteria for either major depression or psychotic disorder at baseline. Major depression and psychotic disorders are included in the concept of severe mental illness of the National Institute for Mental Health as mental disorders that result in serious functional impairment, which substantially interferes or limits one or more major life activities (National Institute of Mental Health, 2019).

Data on the subjective experience of individuals with SMI during imprisonment are scarce and most research has been conducted in high income countries. Qualitative research in long-term imprisoned individuals showed that those with SMI experience more hostility in prison and attribute their suffering to external factors. Whereas, imprisoned individuals without psychiatric disorders described more introspection when experiencing similar stressors (Yang et al., 2009). Qualitative research in the prison context has often been used on selected groups or in order to assess new treatments. A study with male participants described high levels of stress and negative impact of imprisonment on preexisting mental illness as main mental health challenges (Oliffe et al., 2018) whereas other qualitative findings suggest long periods of isolation and negative relationships between staff and prisoners to be associated with poor mental health (Nurse et al., 2003). The participation in qualitative interviews itself can be experienced as beneficial by imprisoned study participants, which might be due to similar positive effects as from storytelling within a context of incarceration (Rivlin et al., 2011; Bove and Tryon, 2018). The results of prison research may be applied in programs to relieve the burden of mental health symptoms and to reduce criminal recidivism.

One of the most important approaches in rehabilitation of prison populations is the *Risk-Need-Responsivity* (RNR) model, that assigns different interventions to specific types of imprisoned individuals (Andrews et al., 1990). Despite its structured approach, the adherence to the principles is poor when applied in the community and better tools for RNR assessment are needed (Dyck et al., 2018). Even though it has been argued, that strength focused approaches such as the *Good Lives Model* do not substantially add to this intervention (Andrews et al., 2011), a focus on resources and interests of the offenders might be promising. Qualitative research data can give insights on strengths and resources of individuals that might be transferred to others according to cultural and sociodemographic context, preferences and types of mental disorder.

Qualitative research data on individuals with SMI within prison context in LMIC may contribute to identify service needs, and to develop policies and services. We conducted a 3 years follow up of imprisoned individuals who had severe mental illness at intake to the prison system in the Metropolitan region of Santiago de Chile to assess the subjective course of their mental health during incarceration.

We aimed to generate in-depth information on the subjective course of SMI during imprisonment in Chile and to identify possible influencing factors.

## Materials and Methods

### Sample

We conducted a follow-up study on 19 individuals with severe mental illnesses at intake to imprisonment. The study consisted of quantitative baseline measures and qualitative 3 years follow-up assessments. The study population is part of a larger quantitative follow-up sample (Gabrysch et al., 2019).

Inclusion criteria were the diagnosis of a severe mental illness (SMI) at baseline and continuous imprisonment until follow-up in a prison facility of the metropolitan region of Santiago de Chile. At baseline, 427 randomly selected prisoners were assessed within their first weeks of imprisonment in three central remand prison facilities of the metropolitan region of Santiago de Chile. The study had shown high rates of SMI (psychosis and/or major depression) (Mundt et al., 2015b).

For the quantitative follow-up all individuals that were assessed at baseline and were still or again imprisoned, were approached, 73 were included in the study (Gabrysch et al., 2019). Among the follow-up sample we selected those who had presented with either major depression, psychosis or both at baseline and approached them for participation in the qualitative assessments. We aimed for *n* = 10 with psychosis at baseline and *n* = 10 with major depression at baseline in order to reach saturation of the data. Twenty-two individuals met the inclusion criteria, three did not respond on the third call and could not be included. We approached *N* = 19 for the qualitative follow-up study and all consented to participate.

Participants were located in five different prison facilities for remand and sentenced prisoners in the metropolitan region of Santiago de Chile: Centro de Detención Preventiva (CDP) Santiago Sur, CDP Santiago Uno, Centro Penitenciario Femenino (CPF) San Joaquin, CPF San Miguel and Centro de Educacion y Trabajo (CET) Semiabierto. The housing infrastructure of the prison facilities in Chile is poor in general. Levels of hygiene are low, warm water is often unavailable. Most facilities can provide access to nursing, a general medical practitioner and psychologists, in exceptional cases also to psychiatric evaluation and treatment.

### Instruments

The MINI Neuropsychiatric Interview was conducted to obtain diagnoses (Sheehan et al., 1998), and the revised version of the Symptom Check List with 90 items (SCL-90-R) was used to quantify mental health symptoms (Derogatis, 1977). The MINI has been used in epidemiological research to establish the prevalence of mental disorders in prison populations in Chile (Mundt et al., 2016). It classifies mental disorders according to the Diagnostic Statistical Manual of Mental Disorders (DSM-IV) and the International Classification of Diseases (ICD-10). The SCL-90-R has previously been used to quantify psychopathological symptom load in prison populations (Gibbs, 1987; Taylor et al., 2010; Ignatyev et al., 2016).

Qualitative data were obtained using semi structured interviews. Standardized questions were applied by the interviewers after shortly introducing the qualitative interview method and selection of the interviewee based on the SMI at baseline.

Do you still have the mental health problem (depression/psychotic disorder) you had 3 years ago when you were committed to imprisonment? In which way do you experience it?
Did you note changes in your mental health during the last 3 years? What changed?
Did you change the patterns of drug and/or alcohol use?
What were factors that influenced these changes?
Did you receive any treatment?
In relation to suicide risk – What influenced you to attempt suicide, what stopped you from committing suicide?

Upon those questions, the interviewees expressed their first thoughts and feelings. The interviewer aimed to deepen the understanding by asking for details regarding timeframe, location and episodic or stable character of the problem. Qualitative interviews were conducted with topic guides, which included preset topics that could either be brought up by the interviewee or introduced by the interviewer. Not all topics needed to be addressed. Topics included psychiatric morbidity, therapy, relationships, life inside prison (visits, police, and legal status), perspectives for the future and general health.

### Procedure

The quantitative assessments at baseline and at follow-up were held by trained psychologists, doctorate students of medicine and a senior psychiatrist. The training a supervision of the field team was conducted by the psychiatrist. The interviews consisted of a structured questionnaire, the Mini-International Neuropsychiatric Interview (MINI) and the SCL-90-R and had a duration of 30–60 min. The procedure of approaching the participants, ensuring confidentiality and obtaining informed consent was equal to the procedure used in qualitative assessments that took place after the quantitative follow-up.

Potential participants meeting inclusion criteria were approached by the prison staff and led to separate rooms for information and consent procedures. To ensure confidentiality, interviews were conducted by members of the research team, in absence of prison staff and in private rooms. The field team consisted of two psychologists, one psychology student and one doctorate student in medicine. They were trained by two senior consultant psychiatrists until satisfying interrater reliability between the interviewers was reached.

Qualitative interviews were held on separate occasions after the quantitative assessments and lasted 20–60 min. Interviews were audio-recorded and contextual notes were taken. Records were transcribed by the interviewers.

Participation in the study was voluntary and could be withdrawn at any time. Written and oral informed consent was obtained from every participant prior to inclusion in the qualitative interview. The study participation was voluntary and independent from all legal issues or other benefits. After having resolved all questions, we emphasized that the consent to participate could be withdrawn at any later point of time without further explanations and without any consequences. Capacity to provide informed consent was deducted from the capacity to sufficiently concentrate on the oral and written study information and to reproduce parts of it in order to show that contents and procedures were understood. No treatments were offered to participants of the study. The study was approved by the institutional ethics review board of the University Hospital of the University of Chile (Acta de aprobación Número 10 del 06 de abril 2016, Comité Ético Científico, Hospital Clínico Universidad de Chile), by the Ministry of Justice (Oficio Número 2478, del 19 de abril 2016, Jefa División Reinserción Social) and by the national prison administration, Gendarmería de Chile (Oficio Numero 671/2016 del 9 Nov 2016, Director Regional Metropolitano Gendarmería de Chile).

### Analyses

We provide descriptive quantitative data on our sample that was analyzed using IBM SPSS Statistics Version 24. Qualitative data were thematically analyzed using a structured six-phase-approach including familiarization, initial coding, searching for themes, reviewing themes, definition of themes and reporting (Braun and Clarke, 2006). Interviews were conducted in order to collect data on the research question – *What is the subjective course of SMI during imprisonment and which factors influence it?* – which provided the theoretical framework for further analysis. The theoretical framework consisted of the three main themes *Subjective course of SMI, Specific course of the disorder (Psychosis or major depression)* and *Influencing factors.* QSR International NVivo Version 11.4.0 software was used for initial coding and quantitative content analysis by identifying the most used words and most referenced categories of codes. The interviews were then coded manually using a theoretical approach with the predefined themes as a base. Consequently, transcripts were analyzed using inductive thematic analysis in order to identify themes and patterns in a *bottom up* way (Hayes, 2000; Frith and Gleeson, 2004). Themes were rearranged and renamed during inductive analyses in order to exhaustively explain the complex coherences of the subjective course of SMI.

Four interviews were independently rated by two senior psychologists, the resulting codes were compared and discussed and reached good inter rater reliability. The remaining interview transcripts were independently rated.

## Results

### Recruitment

We aimed to include 20 participants (10 male and 10 female) of a quantitative 3-year follow-up study who had major depression and/or psychotic disorder at the baseline assessment. Of 22, three did not respond on the third call, none refused to take part in the qualitative assessments. Non-response rate was 14%. We included *N* = 19 participants in the study. All participants completed the qualitative assessment.

### Socio-Demographic and Mental Health Characteristics

Socio-demographic characteristics of the sample are reported in Table 1 as total numbers and as percentage values. All imprisoned individuals were non-migrant Chileans. Of the whole sample *n* = 16 (84%) had at least one child. The legal status of *n* = 15 (79%) individuals was *sentenced*, most, *n* = 8, for drug related crimes. Two female individuals were in nocturnal imprisonment. More than two thirds of the sample, *n* = 13 (68%), reported no occupation within the prison, five (26%) were working and one individual was studying. Study participants had low educational levels. At follow-up 90% received visitors. Nine (47%) reported using prescribed medication on a regular basis, five (26%) had attempted suicide.

**TABLE 1:** Socio-demographic characteristics of people followed-up qualitatively after 3 years in the prison system of Santiago de Chile.

	**Total sample**	
	***N* = 19**	**%**
Median age (minimum/maximum)	29 (21/54)	
**Marital status**		
Married	1	5
Divorced	2	11
Single	14	74
Widowed	2	11
**Number of children**		
0	3	15
1	9	47
2	2	11
3	3	16
5	1	5
6	1	5
any	16	84
**Type of crime**		
Drug related	8	42
Robbery	1	5
Violent crime	5	26
Sexual crime	2	11
Other	3	16
**Type of imprisonment**		
Closed prison	17	90
Nocturnal reclusion	2	11
**Legal situation**		
Sentenced	15	79
Remand	4	21
**Occupation**		
Studying	1	5
No occupation	13	68
Working	5	26
**Educational level**		
ISCED 0	3	16
ISCED 1	10	53
ISCED 2	6	32
**Visits**		
Yes	17	90
No	2	11
**Use of medicaments**		
Yes	9	47
No	10	53
**Suicide attempts**		
Yes	5	26
No	14	74

**ISCED, International Standard Classification of Education.**

DSM-IV diagnoses and suicide risk at baseline assessments at intake to prison and at follow-up are reported in Table 2. Out of *n* = 17 (90%) individuals with major depression at baseline, *n* = 10 (53%) still met the diagnostic criteria at follow-up. The prevalence of psychotic disorders was 53% (*n* = 10) at baseline and 26% (*n* = 5) at follow-up. The prevalence of comorbid anxiety disorders was 79% (*n* = 15) at baseline and 68% (*n* = 13) at follow up. Illicit drug use disorder (IDUD) decreased from 63% (*n* = 12) to 42% (*n* = 8) at follow-up. Prevalence of alcohol use disorder (AUD) was 21% (*n* = 4) at baseline and 16% (*n* = 3) at follow-up. In our sample no case of illicit drug or alcohol abuse was reported, all individuals included in these categories met the diagnostic criteria for illicit drug dependence or alcohol dependence, respectively. At baseline *n* = 15 (79%) individuals met the diagnostic criteria for personality disorders (borderline and antisocial PD), *n* = 9 (47%) at follow-up. High suicide risk decreased from 53% (*n* = 10) at baseline to 32% (*n* = 6) at follow-up.

**TABLE 2:** The prevalence of mental disorders by diagnostic groups and suicide risk among prison populations at baseline and at 3-year follow-up.

	**Baseline**		**Follow-up**	
	***N* = 19**	**Prevalence in %**	***N* = 19**	**Prevalence in %**
**Mental disorders**				
Depression^a^	17	90	10	53
Psychotic disorders^b^	10	53	5	26
Anxiety disorders^c^	15	79	13	68
IDUD	12	63	8	42
AUD	4	21	3	16
PD	15	79	9	47
**Suicide risk**				
Low	3	16	5	26
Moderate	2	11	2	11
High	10	53	6	32

**^*a*^Including major depression, recurrent major depression; ^*b*^including current panic disorder, agoraphobia, social anxiety disorder, generalized anxiety disorder; OCD, obsessive-compulsive disorder; PTSD, posttraumatic stress disorder; ^*c*^including current psychotic disorder, current psychotic mood disorder; PD, personality disorder (including borderline personality disorder and antisocial personality disorder); IDUD, illicit drug use disorder/dependence; AUD, alcohol use disorder/dependence*.*

We included *n* = 10 men and *n* = 9 women. Diagnostic criteria for major depression at baseline were met by *n* = 9 women and *n* = 7 men, *n* = 7 women and *n* = 8 men reported suicidality, *n* = 3 of the individuals with psychotic disorders were female, *n* = 7 were male. The mean GSI (Global Severity Index) of the SCL-90-R as a measure of symptom severity decreased from 2.06 (±0.80) at baseline to 1.62 (±0.89) at follow-up.

### Quantitative Content Analysis

NVivo was used to determine the most used words by the imprisoned individuals which were then sorted into thematic categories. Results of the quantitative content analysis are reported in Table 3. The five most used words were *children, work, drugs, mum*, and *visits.* The most referenced categories with 200 or more quotes per category were *family, police, work, substances, health and illness, everyday life, destiny and fatalism, thoughts about freedom.* Quantitative content analysis was used as a guide for final themes and subthemes.

**TABLE 3:** Quantitative content analysis; word and category frequency.

	**Word and category frequency in all interviews**	**Retrieved from *n* interviews (*N* = 19)**
**Most frequent words**		
Children	227	18
Work	177	17
Drugs	133	18
Mum	117	18
Visits	101	19
**Most frequent word categories**		
Family	556	19
Police	271	18
Work	251	19
Substances	218	19
Health/wellbeing	193	19

### Structure of Themes and Subthemes

**FIGURE 1:**
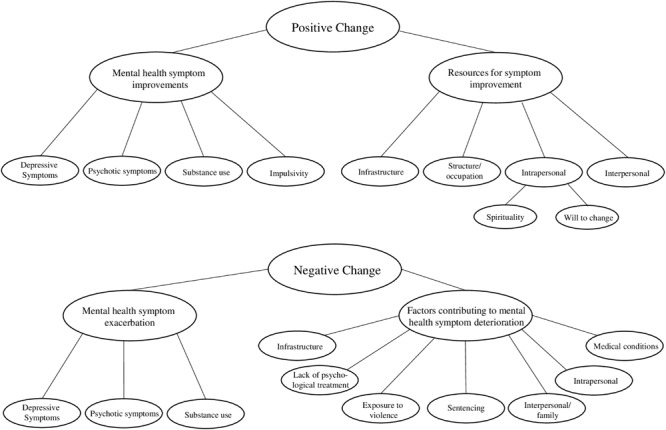
Final themes and subthemes resulting from the inductive thematic analysis.

During the process of inductive analysis, predefined themes that derived from inclusion criteria and hypotheses were modified, in order to outline largely overlapping mental health problems and high rates of comorbidities in the sample. The final analysis and grouping of themes and subthemes is illustrated in Figure 1. The analysis resulted in a separation of *positive change* and *negative change* with subthemes reflecting specific mental health problems and the most influential factors. This way, we aimed to depict comorbidities and the multifaceted influences.

### Positive Change

Ten individuals perceived positive changes during the 3-year follow-up period. Both genders were present in this group, *n* = 6 of them were female, *n* = 4 were male. Selected quotes that refer to improvement of mental health symptoms or resources for that improvement are reported in Table 4.

**TABLE 4:** Quotes and codes for the overarching theme positive change.

**Subtheme**	**Coded for**	**Quote**
**Theme: mental health symptom improvements**		
Depressive symptoms	I will never try to kill myself again	*“When my children saw the scars form my suicide attempt, my soul was hurting and I thought ‘I will never try to kill myself again’.”* F56 *“I don’t want to kill myself anymore.”* F56
		*“It is not like before, I do not want to cut myself, nothing like this.”* F68
	My mood is better and stable	*“I am happier, not getting so angry and my mood is more stable.”* F16
		*“I am motivated, I have energy now.”* M103
		*“I feel euphoric, I take my medicine and I want to do everything.”* F56

Psychotic symptoms	I don’t have hallucinations anymore	*“I was troubled, having so many thoughts about my home, my family, my brothers. I think in one moment my mind just collapsed and I started to see things. Now it is gone.”* M103
		*“I don’t hear the voices anymore, it stopped a year ago.”* F173
		*“I don’t have hallucinations anymore.”* M132
	Everyone sees those “shadows,” it is a part of prison	*“Mostly I see the “dead souls” that move around this prison*… *I see their shadows, but everyone is used to it, we don’t give it much attention.”* M26
		*“I also saw the shadows in prison*… *but that was because someone died before in the bed that now was mine. Scary.”* M6

Substance use	I don’t do drugs anymore	*“Now I don’t have the urge to consume anything, but rather be healthy, be ok to be outside”* M103
		*“I don’t consume and I can help others to change, because I was able to change.”* M54
		*“I did consume (drugs), but now the only thing I consume is tobacco.”* F16
		*“It will soon be 3 years without drugs, but I feel great, I don’t have craving anymore.”* F161 *“I am better now, because I thought and reflected about what drugs are, and how they destroyed my life. Before, I was just thinking about how to get my next high, now, I am thinking about getting out of prison and finding a job as a hairdresser.”* F161

Impulsivity	I am not impulsive anymore	*“Even though complicated situations arise, I learned to control and manage this.”* M54
		*“[I am] good, because I am not walking around with that urge to hit everyone anymore.”* M26
		*“I am not impulsive anymore.”* F56

**Theme: resources for symptom improvement**		
Infrastructure	I am in a nicer prison now	*“I am much better here, there are trees, I see those apartments over there, there (CPF San Miguel) its “Jail” here its “beach” because over there everything is made of iron.”* F105
	I am in a clean, nice ward now	*“I am in a catholic ward now; I asked to be moved here because where I was before there were a lot of drugs, which bothered me. I wanted to be in a calm patio (living area) without drugs*… *here everything is clean; we do not smoke in the dormitories. If you want to smoke during lock-up, you go to the bathroom. And the people here are different, more self-controlled, there is not an immediate conflict if you cross eyes with someone.”* F56
		*“My section is not so terrible.”* F68
		*“I am in Ward 5 now*… *it is the best section in the whole penitentiary.”* M132

Structure/occupation	I feel good because I work/I am occupied	*“I feel good, because I work, because I am active all the time and also because I have been employed here for a while. (…) For two and a half years I worked as a seamstress for the police. When I just got into prison that helped me a lot, kept me occupied, made time go by faster.”* F105
		*“The workshop helps me, because I make money to support my mother.”* F161
		*“I cleared my mind working and studying.”* F173
		*“I gave it (the money) to my wife, so she has money to come visit me.”* M26
		*“I am participating in the wooden mandala workshop that is taking place in our catholic section; I also signed up for school and I have my two duties.”* F56
	Every workshop prison offers can help me, so I do it	*“They gave me all the tools and I took them all. I do everything they tell me, classes, workshops. If they send me to work, I go. If they want to teach me something, I listen because anything that can help me is positive.”* F16
		*“I took part in a workshop on occupational reintegration*… *a workshop where they try to fit you into a field of work; so you can return to society and behave differently*…*work.”* M103
	I have benefits in prison now	*“I can go out, I have benefits now.”* F21
		*“I have little time left, until I get my first Sunday leave.”* F161
		*“I am good now, looking ahead with confidence, working on my good conduct (to get benefits)t, to leave this place, wanting to fight for something*…*”* F56

	I take care of myself now, I have a routine	*“I feel better also because of my self-care, taking care of my room here, I do my things*…*I sleep at fixed hours, I eat at fixed hours, I am orderly, I want to clean my room, wash myself, all those things that meant nothing to me before.”* M256
		*“I am good now, as a fact, I eat. Every day, I have a snack, I have breakfast.”* F16

Intrapersonal -Spirituality	When I feel bad I go to church in prison, it helps	*“I enjoy going to the Holy Mass, I sing with devotion and I like it*… *It helps me, because I believe in god, I know that everything will resolve just fine.”* F56
		*“When I feel bad, I go to church.”* M256

Intrapersonal – will to change	A stay in prison can open your eyes – I really want to change my behavior	*“I think that here they give you an opportunity, if you want you take it. And, if you do not want it, you leave it. There are some (imprisoned individuals) that take it just to get back on the streets sooner, or because they want to go home, or they are bored. And there are others like me, that take it because we really want to change.”* F16
		*“A stay in prison can open your eyes.”* M54
	Maybe it is only in prison that I could change like this	*“Maybe it is only in prison that I could change like this. Not outside with rehabilitation, nor in a center, nor with therapy, nor with a psychologist.”* M54
		*“I think it is within yourself, and if you cannot do it on your own, then you have to ask for help.”* F68
	I want to change	*“I have little time left, so now I am set up to do things right.”* M103
		*“I want to change, and I want to give my son stability and a good future, so that he never ends up here in this place.”* M175
		*“I really want to get out. I plan to work with an old friend when I am out of here.”* M256
	I have thought about things/compared myself to others	*“It is like I am giving myself the last opportunity to change.”* M256
		*“Now, I have grown and thought about things. I do not think about going back to robbing after release.”* M26
		*“There are people worse than me*… *so I see that I am not that bad and that inspires me.”* F105

Interpersonal	When I get visits, I feel good	*“When my son comes to visit I feel good, he gives me energy.”* F173
		*“My depressive symptoms were constant, they only went away on the days of visits.”* M175
		*“The visits make me feel good*… *my daughter comes, my wife*… *it is sad as well, but only for a moment, then it goes away.”* M26
		*“When my kids come to visit, it makes me calm.”* M182
		*“Previously my son did not want to talk to me, he didn’t even visit. Now he is coming.”* F105
	I am a woman in nocturnal reclusion, I am calm because I am closer to my family	*“I am calm now. I can go out on Sundays.”* F16 *“Now I am applying to get weekends off, I can leave every trimester, I spend Christmas with my family, I spent New Year’s Day with my family, everything is really good. Everything is different here*… *it is almost like being completely free. I am already closer to my family, to my children.”* F16
		*“I am happy for having changed the prison facility.”* F21 *“At least we can go out, I just got my trimestral leave approved, soon I can get Sunday leave.”* F21
	My family supports me	*“With the support of my family I knew that I only had to do my time and would go home after I paid (for it)*… *my mum and my brother are those who come*… *once a month or sometimes twice.”* M103
		*“I am inspired, because my son is advancing to fourth grade of middle school with good grades, my family supports me. Those are the things that make me feel better.”* F105
		*“I learned to value the people that are close to me, the ones that I did not value before.”* M256
		*“He (my partner) was the only one to support me.”* F16
		*“I am back in good mood for the last month, I fought with my partner, now she is coming to see me.”* M26
	Now I get along with my family	*“I am more stable, I get along with my son now.”* F105
		*“I see my little nephew and I am happy.”* F68
		*“My good mood came back a month ago, I had a fight with my wife, and now she is coming to see me.”* M26
		*“I was a person that had less friends every day, less family, I felt alone – and that changed.”* M256
	My relationships are better now	*“Well, I think my relationships are better now.”* F21
		*“I was very reluctant to that (building relationships), but that is getting better now.”* M256
		*“When I wake up in the morning, I can greet everyone with a smile.”* M54

#### Mental Health Symptom Improvement

Mental health symptom improvement was reported by interviewees with major depression and/or psychotic disorders at baseline. Out of 19 interviewees *n* = 17 had diagnoses of major depression at baseline. Out of this subgroup *n* = 9 still had major depression at follow-up, and one was newly diagnosed with major depression at follow-up, resulting in *n* = 10 individuals meeting the diagnostic criteria for major depression at follow-up. In the qualitative interviews, symptom improvements in the depression spectrum were reported by *n* = 7 individuals. Especially elevated and more stable mood, more positive thoughts, higher energy levels and the absence of suicidal thoughts, plans or suicide attempts were subjectively important.

The diagnostic criteria for psychotic disorders at baseline were met by *n* = 11 out of 19 interviewees, at follow- up we identified *n* = 5. One of the individuals diagnosed with psychosis at baseline received medical treatment. At follow-up *n* = 4 described an improvement of psychotic symptoms. Especially the absence of hallucinations, both acoustic and visual, was reported by the affected individuals. Several interviewees described the appearance of ‘*shadows*’ or ‘*figures*’ inside prison that tend to come at night, especially to places where people died or were murdered. The imprisoned individuals talk openly about it, and there is an understanding, that everyone sees them which, led us to the conclusion that these are not hallucinations that are part of a psychotic disorder, but rather form part of a mystical prison culture where death and fear play an important role. This was confirmed by the statement that imprisoned individuals ‘*get used to it (the appearances’)* over time.

In qualitative interviews, substance use repeatedly came up as a comorbid disorder that determined the course of mental health. At baseline *n* = 12 met the diagnostic criteria for IDUD, at follow-up *n* = 8. Substance use was described by *n* = 15 individuals as an important part of their past life, *n* = 13 stopped using drugs, one woman described increased drug use and one man referred to his substance use as ‘*non-problematic.*’ We observed that those who stopped using drugs were in the group that could subjectively improve their mental health. Cessation of craving and having had time to reflect the substance use patterns was perceived as helpful.

Less impulsivity was described as an improvement of mental health symptoms that made everyday life much easier, especially building relationships in the prison environment. One man did ‘*not have the urge to hit everyone anymore*’ and therefore felt relieved.

Exploring possible gender differences, we observed that out of *n* = 10 who described improvement of their mental health six were female, four male. Two of the women who experienced improvement were in the facility for nocturnal reclusion which might have had a positive influence. In our sample we could not include any males in nocturnal reclusion, which is more common for women in Chile. Participants of both genders that diminished the amount or stopped consumption of illicit drugs experienced mental health improvement.

#### Resources for Mental Health Symptom Improvement

We defined resources for mental health symptom improvement as factors that were described by the interviewees in connection to wellbeing, subjective improvements, and positive thoughts.

According to those who had the opportunity to change to one of the newer prison facilities, the better infrastructure (an own bed, access to bathrooms, windows, and access to healthcare), views and personal space had a positive influence on mental health. The common term among imprisoned individuals for the newer facilities was ‘*beach*,’ a very figurative word related to relaxation and tranquility. Transfers within the same facility to a cleaner, ‘*nicer*’ section also improved the well-being. Not only cleanliness, but also respect toward smoking and drug bans contributed to a feeling of being safe. Being able to choose the location within the facility according to religion, work group or educational aims seemed to create a positive dynamic.

A structured everyday life and occupation in remunerated work, workshops or studying helped to reach more clarity of thoughts and feelings of worthiness in the imprisoned individuals. One important point was, that it ‘*keeps you busy*,’ as one woman put it. Remunerated work allowed to support the family on the outside and gave the employees the perspective to work in a similar field after release, which reduced anxiety and fear regarding the future. Individuals, who took part in workshops on reintegration into society, perceived them as very helpful. One women referred to take part in ‘*everything they (prison staff) tell me*’ appreciating the offer as well as the own dedication to take and stick to it. The possibility to apply for benefits like Sunday leave, more visiting days etc. that could be gained by good conduct, were incentives for good behavior, abstinence, and self-control. Prison structure allowed, for some individuals for the first time, to implement a routine of hygiene, self-care, nutrition, and order/cleanliness that improved the quality of life compared to the situation prior to imprisonment. Several of the interviewees had an emotionally and economically problematic family background and some even had been living in the streets.

As a strong intrapersonal resource we identified spirituality, specifically the possibility to practice their belief or religion alone or within a community, especially during hard times. The most fundamental resource we called ‘willingness to change,’ which was also based in the individual. Nevertheless, it seemed to be necessary to benefit from external offers and improved settings and might even be a result of imprisonment. As M54 puts it ‘*a stay in prison can open your eyes*.’ It seemed that the distance to the usual environment, the time to think, to self-reflect, the opportunity of occupation and also observing other individuals both as a negative example/warning or motivation all form a special environment inside prison. Reflecting on that led M54 to the conclusion that ‘*it is only in prison that you can change like this.*’ In the field of interpersonal resources for symptom improvement good and supportive contact to close family members was the most dominant aspect that was described. Visits of family members contributed to feeling good and calm. Both men and women experienced an improved relationship to their families as a factor that contributes to better, more stable mood and overall mental health improvement. Women who could change to nocturnal reclusion found the best aspect of it the closeness to their families. Not only the mere fact of seeing family, but also the quality of the relationship was important. A supportive family alleviated the worries about reintegration and how to find a place after release, M103 can ‘*go home*,’ F105 saw her son as ‘*inspiration*’ rather than missing out on his development. Being separated from family, M256 ‘*values people that (he) did not value before.*’ Positive impact was also described from interpersonal relationships with other imprisoned individuals. This was possible due to acquisition of new relationship skills, more stable mood, less aggression, more confidence and better self-control – M54 can ‘*greet everyone with a smile*’ when he wakes up in the morning.

### Negative Change

During the 3 years follow-up period *n* = 6 out of *N* = 19 individuals perceived negative changes, while *n* = 3 described a stable state of strong psychological symptoms. The group of six participants that experienced deterioration consisted of three male and three female individuals. The three individuals that did not perceive any change of their high burden of mental health symptoms included only male individuals. Quotes that refer to exacerbation of mental health symptoms or factors that promoted deterioration are reported in Table 5.

**TABLE 5:** Quotes and codes for the overarching theme negative change.

**Subtheme**	**Coded for**	**Quote**
**Theme: mental health symptom exacerbation**		
Depressive symptoms	I feel sad in prison	*“You will never feel good being here.”* M132 *“As a prisoner nothing can make me feel better.”* M132
	I just want to die	*“I just want to die, I do not know what to do anymore.”* F108
		*“Suddenly I feel depressed, which I never felt before. I want to cut myself, to cry*… *I feel so alone.”* F135
	I have many ups and downs in my mood	*“With ups and downs, because of problems, being locked up, because of things that happen day-to-day.”* M182
		*“My mood was better already, but with all those things that lately happened, it is getting worse again.”* F16
		*“I am happy for 2 days, when I receive visitors and the following day. The rest of the days I am not (happy).”* M175

Psychotic symptoms	I still hear the same things	*“I still hear the same things, I try to distract myself to not think about it, the things I hear, the people that talk [inside my head] are always there.”* M175
		*“All of a sudden I have thoughts, that those thoughts are not mine, it is not my mind. It is rather someone, that has my voice, that says ‘Tell him to shut up already’.”* M31
	Here in prison I started to see things	*“I see things at night. Every night there is a man standing next to me, but he is not dressed in black, he is dressed in cotton. He looks at me and feels my breath.(*…) *I have problems inside my brain, I could just kill myself. I felt depressed before, now I scream, I cry, I scream out loud.*’ F135

Substance use	In prison I can easily consume drugs	*“Here in prison there are more drugs than on the streets. It is easier, closer, you have to move less (to get it.)”* F108
		*“In prison you have the possibility to consume drugs, it seems even easier than outside.”* M6
		*“During those 3 years here I didn’t consume alcohol*… *I almost don’t do cocaine, just sometimes marihuana.”* M175

**Theme: factors contributing to mental health symptom deterioration**		
Infrastructure	I will never feel good in prison	*“How will you get better if you are sentenced for almost the rest of your life*…*”* M178
		*“You won’t feel good being here, even with visitors, work and everything.”* M132
	It is overcrowded – we have three bathrooms for 300 people	*“In Santiago One, we call it “the beach,” I lived by myself in a room. When I was there, I lived in peace. Here in Santiago Sur you have three bathrooms for 300 people and you have to stand in line for hours just to shave”* M182
		*“At 4 pm they close you up no matter the unbearable heat, in a tiny room with five others. You cannot imagine the heat*… *there are more human rights in the streets. Outside the police comes to save a dog that waits too long tied down in front of the supermarket. Here we don’t even have running water, it only works around 3 am.”* M178

Lack of psychological treatment	The psychologist in prison just doesn’t attend me	*“I would really like to talk to someone about it*… *and I would like someone to treat my drug problems, a psychologist or something. Everything is really complicated*…*”* M182
		*“I would like to get therapy or pills to treat my anxiety. Whatever*…*I would like to have professional help, so I do not want to do drugs all the time.”* M6
		*“I need a psychiatrist that can look inside my brain because I see things that I cannot keep on seeing like this*…*”* F135
		*“Is there any chance to get a psychologist? They never want to attend me.”* F108
		*“The only way to save me would be a psychologist, but here the psychologists sometimes just don’t care about you.”* F173

Exposure to violence	The other prisoners want to kill me	*“I do not like to fight, but at the same time, I want to fight. If I do, I lose my nerves, I try to avoid it. But since I have this problem that I cannot sleep and see people, I have the feeling that they are talking close to my ear.”* F135
		*“They just want to kill me inside here*… *now, I do not even go out of my room, I am too scared.”* M31
		*“What affects me are the problems here, within prison, the allegations, the fights.”* M132
	The prison staff hits me and others	*“I was hit yesterday by an officer because I closed the door loudly and I was asking for sleeping pills. Yesterday the police beat up 5 other prisoners that were fighting, but you should see how they hit them – worse than dogs.”* M178
		*“I think the police staff has problems at home, and they take it out on us.”* M26

Sentencing	I was sentenced without proof and will be free when I am sixty; I feel terrible, feeling of injustice	*“Now I am a prisoner because of a law, not because of justice; because of presumptions*… *all just heard, nothing proven*…*“What a future! I will get out when I am 60 years old! Who will give me work*… *I cannot even think about having a family*…*”* M178

Interpersonal/family	I was separated from my partner, my world crashed	*“This (separation of romantic partner) really changed my mood. I feel like my whole world crashed.”* M6
	I cannot sustain my family	*“I need to sustain them financially, but I cannot.”* F173
		*“I can do nothing for my children and grandchildren.”* M182
		*“No, I can’t do anything of what I should do (to support them)?”* F16
	I feel bad, when my daughter does not recognize me	*“I feel bad, when my daughter comes, because she does not recognize me. I will be out when she is 15, what is she going to say to me? That I abandoned her at age five.”* F108
	I lost my kids due to imprisonment	*“I have a 4 years old boy*… *but I lost him because of all the time in prison. I could not look after him. They took it away. It is painful for me to have lost him. They gave him into adoption.*…” F135
		*“I suffer a lot here. I missed my daughters’ whole childhood.”* F21
		*“I can’t be calm; I can’t stop thinking about her*… *my daughter.”* M6
	I don’t want that my child comes to see me in prison	*“I don’t want that my daughter comes here.”* F135
		*“I do not want my daughter to come visit. It is my crime, not hers.”* F108
	I am afraid and depressed that I don’t know how to be a dad	*“Being here, not able to see her (my daughter)*… *I missed all of her childhood, her first steps, her first teeth, her jokes, all this*… *depresses me, not knowing how to be a dad.”* M31
	When I fight with my loved ones, I feel bad/rejected	*“When I fight with my daughter, I feel bad, decimated, destroyed.”* M182
		*“I got arrested and now my wife does not want to hear anything from me.”* M6

Intrapersonal	I am insecure/afraid about my role outside of prison	*“I barely have any time left here (in prison), only 6 months, so there are so many things coming up. There is so much information about being outside, so I am really stressed about it.”* F16
		*“I do not know what waits for me outside.”* M31
		*“I am afraid of going back to the streets, going back to my old ways. All those months for nothing. Because going out and doing the same things just lead back here, or I do something worse: a robbery or I kill someone for drugs*…*”* M6

Medical conditions	I have HIV	*“What burdens me most is having HIV*… *I feel bad for having HIV, this worries me most. Imagine my son is already grown up. How will I tell him?*… *Or maybe he already knows.”* F173
	I was a victim of abuse before I went to prison	*“My stepfather abused me and I got pregnant. My mom pushed me down the stairs, she wanted the child to die and it did*… *Even if I have to spend the rest of my life in prison, they will have to pay for the pain they caused me.”* F108
	I had mental health problems before coming to prison	*“Prior to getting arrested, I was in mentally ill.”* M54
		“*I was mentally troubled from living in the streets.”* M103
		*“I came to prison with those symptoms, of not wanting to live, with the drugs, not being able to cheer myself up.”* M256
		

#### Mental Health Symptom Deterioration

At follow-up *N* = 10 individuals met the diagnostic criteria for depression, of which one had not had major depression at baseline. In qualitative interviews persisting or new symptoms depression, sadness linked to being imprisoned, suicidal thoughts or the wish to die as well as unstable or bad mood were described. F108 did ‘*not know what to do anymore*’ feeling left alone and hopeless.

At follow-up *n* = 5 still met the diagnostic criteria for psychotic disorders, *n* = 7 described psychotic symptoms during the follow-up period in the qualitative interviews. Some individuals in this group had auditory hallucinations, *n* = 4 reported to hear voices. This was new and frightening to some, and had manifested for the first time during imprisonment. Others continued to hear voices during imprisonment, like M175 ‘*still hears the same things.*’ Apart from the visual appearances that are a part of prison culture, F135 started to ‘*see things*’ that burden her to the point of having suicidal thoughts.

For some, substance use and especially alcohol consumption decreased during imprisonment. Our data suggest that, some took the personal decision to use imprisonment as an opportunity to be abstinent. The individuals who continued using drugs ‘*have the possibility to consume drugs, it seems even easier than outside*’ for M6. Also, normalizing the use of drugs that were perceived to be less dangerous can be considered a possible problem for mental health, like M175, who stopped drinking and now ‘*almost doesn’t consume cocaine, just sometimes marihuana*’.

#### Factors Contributing to the Deterioration of Mental Health Symptoms

We defined factors contributing to mental health symptom deterioration as aspects that were described by the interviewees in connection to feeling bad, symptom exacerbation, and negative thoughts. Some interviewees stated, that, no matter how new the facility, how many visitors they receive, they will never feel good in prison, which probably derives from the social construct ‘prison’ as a punishment where no one is supposed to feel good. Overcrowded prison facilities and bad infrastructure contributed to poor mental health. Participants referred to ‘*share a bathroom with 300 people*’ (M182), ‘*have no running water*’ (M178) and the feeling of being deprived of human rights. The constant fear of being moved to the worst parts inside prison facilities interferes with focusing on rehabilitation, as everyone has to look after his own survival. The lack of medical and psychological services was also related to overcrowding. Even though all participants had severe mental illness at intake, most did not receive psychological or psychiatric attention. This treatment gap and the unfulfilled expectation to receive psychological or psychiatric treatment had negative effects on mental health symptoms. Individuals with substance use, anxiety and hallucinations felt neglected by prison health professionals and believed that ‘*therapy or pills. Whatever.*’ (M6) might help, or as F173 puts it more drastically ‘*the only way to save me would be a psychologist.*’

Exposure to violence within the prison context was a relevant factor for ill mental health, especially for male individuals. Nevertheless, it also was an issue for some women in prison. Especially violent and non-violent conflicts between imprisoned individuals were described regardless of gender. F135 said ‘*if I do it (fight), I loose my nerves*’ so she tried to avoid it, which was difficult for her since acoustic hallucinations gave her the feeling of being in an even more hostile environment. Untreated psychiatric disorders and crowding increased the potential for conflicts. In some cases, mainly in the biggest all-male facility, it is well known that the fights between imprisoned individuals, or gangs could quickly escalate and even lead to homicide. M31 had fear and expressed ‘*They just want to kill me here*… *now I do not even go out of my room, I am too scared.*’ Also physical violence from prison staff was an issue for many.

One interviewee, M178, was affected most by his sentence, as he strongly insisted on being innocent. He felt terrible and a sense of injustice. His thoughts about his future were sinister as he would get released at the age of 60 years, without any prospects to find work or build a family.

Imprisoned individuals with SMI had the most relevant relationships with their close family members. Separation from romantic partners and the inability to financially and morally support their families from within prison were factors contributing to feeling lonely and worthless. Parenting roles were lost or altered due to imprisonment, in some cases interviewees were sad having missed out on the entire childhood of their children or not being recognized by their children during prison visits. One daughter was given into adoption, when the mother (F135) was sentenced. Others were too ashamed to meet their children during imprisonment. M31 did not express the feeling of loss, but was afraid that he never learned how to be a dad and was depressed by the thought of failing in this role after release. We identified conflicts with close friends, family and romantic partners as a potential trigger for feeling depressed mood and rejection. There was a sense of helplessness knowing that conflicts could not be solved until the next visit or potential phone call.

The main intrapersonal factor contributing to mental health symptom deterioration was the insecurity about life after release. There was uncertainty about ‘*how it will really be*’ that evokes feelings of anxiety. The lack of self-confidence caused fear of ‘*going back to the old ways and (to commit) robbery or kill someone for drugs*…’.

As a burden brought from the outside to the prison context, we identified prior medical conditions or trauma to have negative effects on mental health. The biggest problem was ‘*having HIV*’ for F173, which made her feel bad and guilty, also because she did not know how to tell her son. F108 was a victim of sexual abuse by her stepfather and was determined to take revenge, leading to statements such as ‘(…)*even if I will have to spend the rest of my life in prison.*’ Those experienced indicate, that individuals that had experienced mental health problems prior to imprisonment perceived a deterioration of their symptoms during incarceration.

Exploring possible gender differences, it is important to note, that the three women who experienced deterioration of their mental health all had traumatic experiences. One was raped, one had her children taken away by welfare authorities and one was suffering under the stigma of an HIV infection. The male participants that described deterioration brought up partner conflict and problems to fulfill their roles as fathers.

### Frequency Analysis of Final Themes

Qualitative analyses resulted in four themes and 19 subthemes with 45 codes based on 118 quotes from 19 interviews. The number of codes and quotes for each subtheme is reported in Table 6. This quantitative content analysis was applied in order to identify which factors that influence the subjective course of mental health were addressed by a major part of participants.

**TABLE 6:** Frequency analysis of themes and subthemes.

**Subtheme**	**Number of codes**	**Number of quotes**	**Number of participants (*N* = 19)**
***Overall***	**45**	**118**	**19**
**Theme: mental health symptom improvement**			
Total in theme	6	19	10
Depressive symptoms	2	6	4
Psychotic symptoms	2	5	5
Substance use	1	5	4
Impulsivity	1	3	3

**Theme: resources for symptom improvement**			
Total in theme	16	49	14
Infrastructure	2	4	4
Structure/occupation	4	12	9
Intrapersonal – Spirituality	1	2	2
Intrapersonal – will to change	4	10	8
Interpersonal	5	21	11

**Theme: mental health symptom exacerbation**			
Total in theme	6	14	8
Depressive symptoms	3	8	7
Psychotic symptoms	2	3	3
Substance use	1	3	3

**Theme: factors contributing to mental health symptom deterioration**			
Total in theme	17	36	14
Infrastructure	2	4	3
Lack of psychological treatment	1	5	5
Exposure to violence	2	5	5
Sentencing	1	1	1
Interpersonal/family	7	13	8
Intrapersonal	1	3	3
Medical conditions	3	5	5

## Discussion

### Main Findings

The prevalence of major depression decreased from 90% (*n* = 17) to 53% (*n* = 10), and of psychotic disorders from 53% (*n* = 10) to 26% (*n* = 5) between baseline and the 3-year-follow-up. Ten individuals described a subjective improvement in mental health, while six experienced deteriorations and three did not perceive any change of their mental health. As resources for mental health improvement we identified: good prison infrastructure, structured daily activities and work, intrapersonal factors such as spirituality and willingness to change, and supportive relationships as an interpersonal resource. Factors contributing to mental health symptom exacerbation in prisoners with SMI were: old and overcrowded facilities, the lack of psychological treatment, exposure to violence, interpersonal conflicts, sense of injustice related to the sentence, intrapersonal insecurity and preexisting medical conditions. Quantitative content analyses showed that family, especially children, occupation and drugs had key importance for the subjective course of severe mental illness in prison.

### Strengths and Limitations

The strength of this investigation is the combination of objective assessments and subjective reports on the course of SMI during imprisonment in a Latin American context. All participants had similar socio-economic backgrounds and are of both genders. We also had the opportunity to include imprisoned individuals on remand as well as sentenced individuals with both short and long remaining length of stay.

A limitation of the study was the relatively small sample size. However, we believe to have reached satisfying saturation of data. Nevertheless, there may be potentially more individual factors to improve or deteriorate mental health conditions. Another limitation might be the possible underrepresentation of very severe mental illnesses such as acute psychoses due to the exclusion criteria at baseline. We included individuals who were willing to share their experience, thus the mental health symptoms and views of the more reserved and withdrawn individuals might not be fully covered.

Furthermore, we want to emphasize, that this was a qualitative observational study that describes factors that subjectively influenced mental health and, therefore, strong casual conclusions cannot be drawn from the results.

### Interpretation and Comparison With the Literature

Our study provides insight on subjective experiences of living with SMI during imprisonment and identifies factors for improvement as well as for deterioration of those conditions. The factors range from external influences such as the quality of infrastructure in the prison facility to internal factors such as the willingness to change.

On average, symptom improvement of mental health during imprisonment has been shown by various studies both from western high income countries as well as from low and middle income countries from symptom levels higher than typically seen in community samples (Walker et al., 2014; Baier et al., 2016; Dirkzwager and Nieuwbeerta, 2018; Gabrysch et al., 2019). A qualitative study with incarcerated women described that with regard to preexisting or new mental health problems a lot of positive experiences of support within prison were reported (Caulfield, 2016). In our sample, *n* = 10 individuals (52%) experienced such improvement. Suicide risk in prison populations was found to be higher than in the general population (Fazel and Benning, 2006; Fazel et al., 2011). Participants in this study reported how they controlled the risk to commit suicide. These individuals also reported an improvement of depression symptoms, which according to a typology of prisoners making near lethal suicide attempts were not the ones that are at the highest risk of attempting suicide (Rivlin et al., 2013).

Our sample showed high rates of comorbid substance use disorders, which is in line with the literature (Fazel et al., 2006; Fazel et al., 2017; Mundt et al., 2018). A part of our sample reported to have reduced substance or completely stopped substance use, which suggests, that imprisonment can be an opportunity for substance abuse treatment or rehabilitation. Data from Portugal indicated that incarceration had a positive influence on the health of individuals with drug addiction, but not for individuals with other mental health problems (Alves et al., 2016). There is good evidence for opioid-substitution programs in prisons (Fazel et al., 2016). However, the most prevalent drugs are marihuana and cocaine products in Latin American prisons (Mundt et al., 2018). Findings from a qualitative study showed that the implementation of drug use treatment programs should acknowledge the complex social environment of the imprisoned individuals (Snell-Rood et al., 2016). Imprisoned individuals with comorbid mental health and substance use disorders were likely to have negative post-release outcomes and high prevalence of relapse, but the underlying mechanisms are still to be investigated (Johnson et al., 2013). Most of those using drugs during imprisonment had preexisting substance use problems; one found it even easier to purchase illicit drugs in prison than in the street. Our study supports the findings, that the ban of substance consumption seemed to work for alcohol, but other drugs were available and used in most prisons (Mundt et al., 2018).

One factor related to subjective improvement of mental health in prison was in line with results reported from quantitative research: Meaningful occupation (work or school) had the strongest correlation with symptom scores in the quantitative 3-year-follow-up study from Chile (Gabrysch et al., 2019). In this study, the six individuals, who had work, reported direct positive effects such as a clearer mind and indirect effects such as being able to send money to their families. Given the positive effects on mental health, less recidivism upon release and the preference of most people to work over doing nothing, the current work offers in prison context are unsatisfactory (Saylor and Gaes, 1992; Batchelder and Pippert, 2002; Vacca, 2004). We identified spirituality as a factor that promotes improvement of mental health symptoms. Religion and spirituality during incarceration has been associated with lower frequency and severity of depressive episodes and reduction of incidents and sanctions in a review (Eytan, 2011). Family and positive relationships were identified as protective factors for mental health in this study. This could be consequence of a cultural value known as *familism*, a term used to describe the importance of extended family ties in Latin American cultures, as well as the strong identification, attachment and loyalty of individuals with their families (Castillo and Cano, 2008). Being able to sustain a caring role in the family was also shown to promote positive feelings in women during imprisonment (Sims, 2013).

We aimed to include both individuals that showed improvement as well as deterioration on the quantitative assessment tools used for the follow-up study. Six individuals reported deterioration and three had not perceived any change at all. Interestingly, those who did not perceive any change showed objective improvements in the quantitative assessments such as reduced substance use and absence of hallucinations. A possible explanation for this dissonance might be the limited level of self-reflection and low educational level in the sample, so that not all changes were subjectively perceived, remembered and addressed. The course of major depression during imprisonment points to symptom improvement, with around 50% remission during imprisonment (Zamble and Porporino, 1990; Hassan et al., 2011; Walker et al., 2014; Baier et al., 2016). Nevertheless, a substantial number of individuals experienced major depression during incarceration. Those individuals need adequate interventions that also address mood swings and suicidality. Those symptoms were reported by the individuals in our study who reported deterioration of mental health (Bukh et al., 2013). There are few studies on psychotic disorders in prison, but preliminary findings suggest that negative symptoms such as poverty of speech and blunted affect improve over time (Blaauw et al., 2007). In our study, we show experiences of ongoing (acoustic) hallucinations or thought insertion as well as newly reported hallucinations that started during imprisonment. This might be related to the lack of treatment in this Latin American context compared to the Dutch study that showed improvement in psychotic prisoners with treatment rates of about 80% (Blaauw et al., 2007). This qualitative study and the 3-year quantitative follow-up study from Chile suggest that the course of psychosis in prison shows relatively small and inconsistent symptom improvements. Screening for psychosis at intake and subsequent treatment should be established as routine care (Gabrysch et al., 2019). A qualitative study from France found that long-term imprisoned individuals with SMI attribute their suffering to external circumstances (Yang et al., 2009). We also observed in participants of this study that they attributed mental health problems to external factors such as bad infrastructure, lack of treatment, exposure to violence and bad relationships. Our sample also reported psychological distress related to bad infrastructure, old facilities and overcrowding- conditions that are not in line with the United Nations Standard Minimum Rules for the Treatment of Prisoners (Un Office on Drugs and Crime, 2015). Access to mental and physical health services is a key motivating factor for behavioral changes, and imprisoned individuals actively seek those services (Abad et al., 2013; Sims, 2013). Mental health service and substance use treatments were identified as areas of greatest health needs in another prison based study (Kipping et al., 2011). Data from a female prison showed many positive experiences of mental health support within prison (Caulfield, 2016), the importance of health care and support is emphasized by a study from Portugal which found that incarceration itself had no beneficial effects on women’s mental health (Alves et al., 2016). Interestingly, a British study found, that changes to psychotropic medication management upon entry to prison had negative effects on relationships with prison health staff, disrupted preexisting self-medication practices, discouraged patients to take self-responsibility and may hinder good mental health care in prison contexts (Bowen et al., 2009). Prison mental health care is complex and it may not be sufficient to copy methods from community care.

Exposure to violence was mainly addressed by male individuals during imprisonment, but also present in the female prison context. Our findings, that negative relationships among prisoners and with prison staff have negative impact on mental health, are in line with qualitative data from England (Nurse et al., 2003). Physical violence by prison staff that was described by interviewees in our study raises human rights concerns. The finding that violence by prison staff toward prisoners may impact on recovery was appalling, but not completely unexpected (Almanzar et al., 2015) and merits further research into the type and prevalence in order to improve prevention strategies.

The deteriorating factor with the highest number of quotes was *interpersonal/family*, which underlines the importance of family for imprisoned individuals with SMI. Apart from the separation from intimate partners, the separation from minor children was a main influencing factor, which has also been previously described (Kruger et al., 2017). Distress and negative course of mental health have been shown especially for mothers whose needs were not addressed in the prison context. To support imprisoned mothers with SMI an individual treatment approach at reentry to the community is necessary (Poehlmann, 2005; Arditti and Few, 2008; Few-Demo and Arditti, 2014). The children of incarcerated parents also need to be addressed in treatment plans, as they have high risks of developing antisocial behaviors. Facilitating contacts with children in the prison context as well as treatment and care of the children has to be addressed by policymakers (Poehlmann et al., 2010; Shlafer et al., 2012). In our study, concerns and fear about life after imprisonment were reported from individuals with mental health symptom deterioration. This fear may be justified, as release is often poorly planned and former prisoners are frequently left alone. In the early period after release formerly imprisoned individuals have an elevated risk of death, especially by drug overdose, cardiovascular disease, homicide, and suicide (Binswanger et al., 2007, 2011; Moller et al., 2010; Zlodre and Fazel, 2012; Spittal et al., 2014).

Male and female participants in our sample described similar factors that influenced their course of mental health. According to the literature, female gender is an advantage when recovering from SMI, in our sample 60% of those who experienced improvement were females (Schön, 2010). Especially in a Latin American prison context gender based violence should be taken into account as a risk factor for mental health deterioration (World Health Organization, 2000). Sexual violence and traumatization are a problem for most women that are imprisoned in a Latin American country (Treacy, 1996). In our sample one women shared her trauma of sexual violence, it is very likely that others preferred not to share their experience. More research and support for victims of gender based violence is necessary.

Risk of reconviction is especially high for individuals with SMI (Baillargeon et al., 2009). Improving mental health care in prisons could reduce reoffending and would not only help the imprisoned individuals, but also have larger benefits for the society by reducing crime and positive socioeconomic effects (World Health Organization, 2007). Different models of rehabilitation for criminal offenders have been developed. They either focus on symptom improvement such as the theory of *recovery* or on the criminological outcome of less reoffending, such as the theory of *desistance* (Best, 2019). In the theory of *recovery*, used in psychiatric research, the mentally ill offenders define, when they reach the goal of *recovery.* It is typically reached, when the control of psychological symptoms and the ability to participate in everyday life is reestablished (White, 2007). The theory of desistance has socioeconomically desirable outcomes that can be reached focusing on the individual strengths (Colman and Laenen, 2012).

Our research mainly focused on the outcomes proposed in the theory of *recovery*, nevertheless the results of this in-depth qualitative research may also inform interventions following the *desistance* theory identifying individual factors that strengthen the rehabilitation process (Farrall and Calverley, 2006; Maruna and Mann, 2019). The same factors identified to improve the course of mental health within the prison context – good infrastructure, structure and occupation, intrapersonal resources and supportive relationships – could also reduce recidivism. Our findings could support resource based interventions to reduce the risk of reoffending such as the *Good Lives Model* by giving insight to particular interests, abilities, and aspirations of mentally ill offenders in a Chilean prison context (Netto et al., 2014). Future research should focus on identifying starting-points for a structured improvement of mental health care in prison settings and the development of rehabilitation programs.

Even though prisons may be the wrong place to be for people with SMI and prisons are poor settings for mental health treatment provision, in the unforeseeable future, many people with major mental health problems will be imprisoned in LMIC (Jack et al., 2018). In conclusion, this study has identified factors that may be related to SMI during imprisonment that could be further tested in future quantitative and intervention research. Our findings from this study might help to inform prison service development in order to protect the rights and improve the outcomes of imprisoned people with SMI in Chile and in other South American countries.

## Data Availability Statement

The datasets generated for this study will not be made publicly available. Our dataset contains qualitative interviews of one hour duration, that include details about family, type of crime, gender, age, and health conditions that might lead to identification of vulnerable individuals. We therefore cannot provide access to this data. Quantitative data on our sample is available by request.

## Ethics Statement

The studies involving human participants were reviewed and approved by University Hospital of the University of Chile (Acta de aprobación Número 10 del 06 de abril 2016, Comité Ético Científico, Hospital Clínico Universidad de Chile) Ministry of Justice (Oficio Número 2478, del 19 de abril 2016, Jefa División Reinserción Social) National prison administration, Gendarmería de Chile (Oficio Numero 671/2016 del 9 Nov 2016, Director Regional Metropolitano Gendarmería de Chile). The patients/participants provided their written informed consent to participate in this study. Written informed consent was obtained from the individual(s) for the publication of any potentially identifiable images or data included in this manuscript.

## Author Contributions

AM contributed to the conception and design of the study. CG, CS, and CB collected the data. CS, CB, and CG performed the qualitative data analysis. AM and CG wrote the first draft of the manuscript. All authors critically revised the manuscript and approved the submitted version. AM supervised the study.

## Conflict of Interest

The authors declare that the research was conducted in the absence of any commercial or financial relationships that could be construed as a potential conflict of interest.

The reviewer YI declared a past co-authorship with one of the authors AM to the handling Editor.
